# Contrasting Leaf Trait Responses of Conifer and Broadleaved Seedlings to Altered Resource Availability Are Linked to Resource Strategies

**DOI:** 10.3390/plants9050621

**Published:** 2020-05-13

**Authors:** Yan-Li Zhang, Barbara Moser, Mai-He Li, Thomas Wohlgemuth, Jing-Pin Lei, Christoph Bachofen

**Affiliations:** 1 Research Institute of Forestry, Chinese Academy of Forestry, Beijing 100091, China; zhangylde@126.com (Y.-L.Z.); leijp@caf.ac.cn (J.-P.L.); 2 Forest Dynamics, Swiss Federal Research Institute for Forest, Snow and Landscape Research WSL, CH-8903 Birmensdorf, Switzerland; maihe.li@wsl.ch (M.-H.L.); thomas.wohlgemuth@wsl.ch (T.W.); 3 Key Laboratory of Geographical Processes and Ecological Security in Changbai Mountains, Ministry of Education, School of Geographical Sciences, Northeast Normal University, Changchun 130024, China; 4 Environmental Sciences and Engineering, École polytechnique fédérale de Lausanne EPFL, CH-1015 Lausanne, Switzerland; christoph.bachofen@epfl.ch

**Keywords:** foliar functional traits, light, nutrients, drought, environmental change, evergreen conifers, deciduous broadleaves

## Abstract

(1) Understanding tree seedling responses to water, nutrient, and light availability is crucial to precisely predict potential shifts in composition and structure of forest communities under future climatic conditions. (2) We exposed seedlings of widespread Central European tree species with contrasting leaf habit, deciduous broadleaves (DB) and evergreen conifers (EC), to factorial combinations of manipulated precipitation (100% and 50% of ambient), shade (40% and 60% of full sunlight), and nutrient availability (low and high NPK), and measured specific leaf area, C/N ratio, soluble sugars, starch and non-structural carbohydrate concentration, and δ^13^C of the leaves. (3) We found contrasting effects of water and nutrient availability on foliar traits of the two species groups: EC exhibited higher tolerance to low resource availability but also less plasticity in foliar traits, which is congruent with a “slow” resource strategy. In contrast, foliage of DB reacted particularly to altered nutrient availability, corresponding to a “fast” resource strategy with high foliar plasticity and rapid adjustments to resource fluctuations, commonly adopted by species with high growth rates. (4) We conclude that DB will respond to environmental change with foliar acclimation, while EC will either tolerate, to some extent, or shift their distribution range in response to environmental change.

## 1. Introduction

Changes in water availability are expected to shape the future distribution of tree species in Europe [1,2,3]. Foliar physiological and morphological responses to drought have thus been studied extensively [4,5,6]. Similarly, soil nutrients can influence tree carbon uptake [7,8], limit forest productivity [9], and consequently affect species composition, production, and services of forest ecosystems [10]. Since soil nutrient availability affects plant growth and biomass allocation, it is likely to influence plant performance under drought [11]. Vice-versa, water availability alters soil nutrient status by changing ion mobility and microbial activity [12]. It is, however, still unclear whether high levels of soil nutrients aggravate or mitigate drought stress for trees [11,13]. As both water and nutrient availability affect foliar photosynthetic performance, their effect on trees will be modified by light intensity. For instance, in tree canopies, where water and light availability show large vertical gradients (e.g., [14]), the distribution of nitrogen to different compartments of the photosynthetic machinery is affected by both water and light availability [15,16,17,18]. Moreover, increased light availability may mitigate drought stress of light-demanding tree species by alleviating carbon investment trade-offs between water and light capturing plant parts [19]. Experiments testing the limiting effects of water and nutrient availability are scarce (but see: [13]), and co-limitations of more than two resources are rarely studied (but see: [20,21]). Since foliar resource strategies of trees are linked to resource availability, threefold interacting effects of water, nutrient and light availability on growth performance might differ between species with contrasting leaf traits.

The performance of a tree species under different environmental conditions is determined by its functional traits [22,23,24,25]. At the foliar level, plants modify their morphological, biochemical, and physiological traits in order to optimize resource use for photosynthesis at a given resource availability [26,27]. Together, the functional traits make up a species’ resource strategy [28]. Biophysical constraints among traits result in trade-offs, so that single traits are not independent from each other, but are correlated in what has been described as a trait continuum [22,29]. This suggests that multiple traits are adaptive in association, i.e., environmental conditions do not select for single traits but a “set of traits” [30] or “syndrome” [31]. A combined set of trait values can be advantageous in a particular environment and thus result in a successful ecological strategy [31]. Hence, in addition to biophysical constraints, plants underlie trade-offs in different aspects of their life-history strategies [32]. Similar to trait continua, life-history strategies can be characterized along life-history spectra, such as the recently proposed fast–slow life-history spectrum, which applies to traits of the entire plant (foliage, stem, roots; [22]). Accordingly, a fundamental trade-off between competitive resource acquisition (“fast” life-history strategy, i.e., quick growth and high competitive strength), and resource conservation (“slow” strategy, i.e., low mortality under low resource availability) determines how a plant performs under a given set of environmental conditions [22,33,34]. For instance, plant species with high foliar nitrogen (N) content, high specific leaf area (SLA), and high photosynthetic capacity (Amax), e.g., deciduous broadleaves, have shorter foliar life span than plants with low foliar N, low SLA and low Amax, e.g., evergreen conifers [35,36]. While the advantages of a “fast” strategy are to make efficient use of high resource availability and thus outcompete “slow” plants, the advantage of the “slow” strategy is to survive conditions of low resource supply with low growth capacity [22,30,34,37]. Functional traits are therefore not independent but determine as an ensemble how a species performs under a given set of environmental conditions [22] or how it reacts to altered resource availability [26,31,38]. For instance, plants using the “slow” strategy may generally be more drought tolerant [22], more shade tolerant due to lower respiration rates under low light availability, and more tolerant to low nutrient availability [34]. Conversely, plants applying a “fast” strategy will suffer in any kind of resource poor environment, because their high investments in an efficient resource acquisition system will not pay off [22].

Trait responses to changes in resource availability have been studied across many tree species and biomes [39,40,41,42,43]. However, experiments that compare leaf trait responses of species with contrasting life-history strategies to simultaneous changes in water, nutrient, and light availability are lacking [30]. Evergreen conifers and deciduous broadleaves evolved in different evolutionary times. Accordingly, they developed characteristics from opposite sides of the life-history spectrum [22,29,44] and are thus expected to differ in their response to changes in environmental conditions [45,46]. While evergreen conifers (EC) have long-lived foliage associated with low potential growth rate but sustained growth under conditions of low resource availability, the leaves of deciduous broadleaves (DB) are short-lived, which is congruent with a ‘fast’ life-history strategy including competitive resource acquisition and fast growth rates [47]. Here, we aim at understanding how changing the availability of three major resources for plants—water, light, and nutrients—affects the foliar traits of seedlings of tree species with contrasting resource strategy use: “slow” evergreen conifers (EC) and “fast” deciduous broadleaves (DB). We conducted an experiment with seedlings of tree species widely occurring in Central European forests (four EC and four DB). The seedlings were grown for three years in mesocosms subjected to factorial combinations of manipulated precipitation (100% and 50% of ambient), shade (40% and 60% of full sunlight), and nutrients (low and high nutrient availability). We measured various foliar morphological and biochemical traits to better understand responses of trees to environmental changes. We hypothesize that plants with a “fast” resource strategy (DB) will show stronger responses to changes in resource availability (water, nutrients, light) than plants with a “slow” resource strategy (EC) while the latter will have an overall higher tolerance to low resource availability.

## 2. Results

### 2.1. Evergreen Conifers

**Figure 1:**
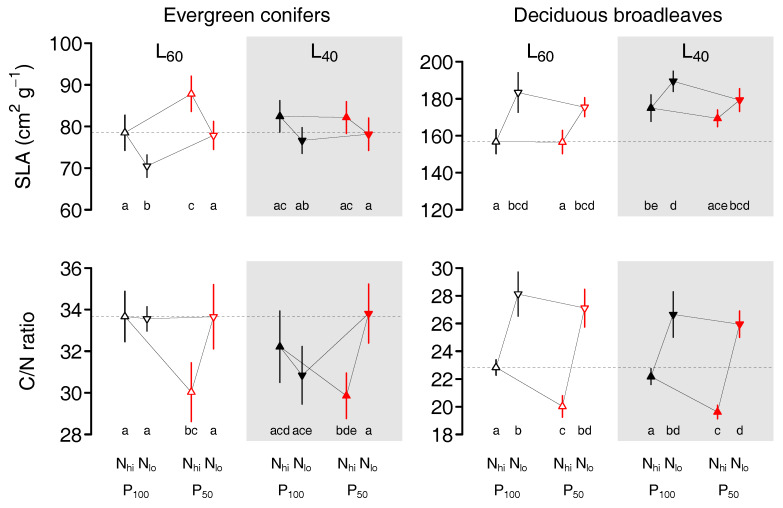
Specific leaf area (SLA) and the C/N ratio (means ± SE) of the foliage of evergreen conifer and deciduous broadleaved seedlings grown for three years under fully crossed combinations of ambient (P_100_, black symbols) or reduced precipitation (P_50_, red symbols), high (L_60_, open symbols) or low PAR (L_40_, filled symbols), and high (N_hi_, upwards triangle) or low (N_lo_, downwards triangle) soil nutrient availability. The dashed line indicates foliar traits under optimal resource availability, i.e., L_60_ × P_100_ × N_hi_. Different letters below the means indicate significant differences between treatment combinations within species group (Tukey adjusted least square means).

The specific leaf area (SLA) of evergreen conifers (EC) was affected by both precipitation and nutrients, but the seedlings responses depended on light availability (Table 1). Under high photosynthetically active radiation (PAR), seedlings grown at reduced precipitation (L_60_ × P_50_) showed higher SLA compared to seedlings grown at ambient precipitation (L_60_ × P_100_), while seedlings in mesocosms with reduced nutrient availability (L_60_ × N) formed needles with lower SLA than those in high nutrient ones (L_60_ × N_hi_, Figure 1). In contrast, under low PAR (L_40_), SLA was intermediate and the needles showed no responses to either precipitation or nutrient availability. The foliar C/N ratio of EC seedlings was lower under the combination of reduced precipitation and high nutrient availability (P_50_ × N_hi_) compared to reduced precipitation and low nutrients (P_50_ × N_lo_,) in both shading treatments.

**Figure 2:**
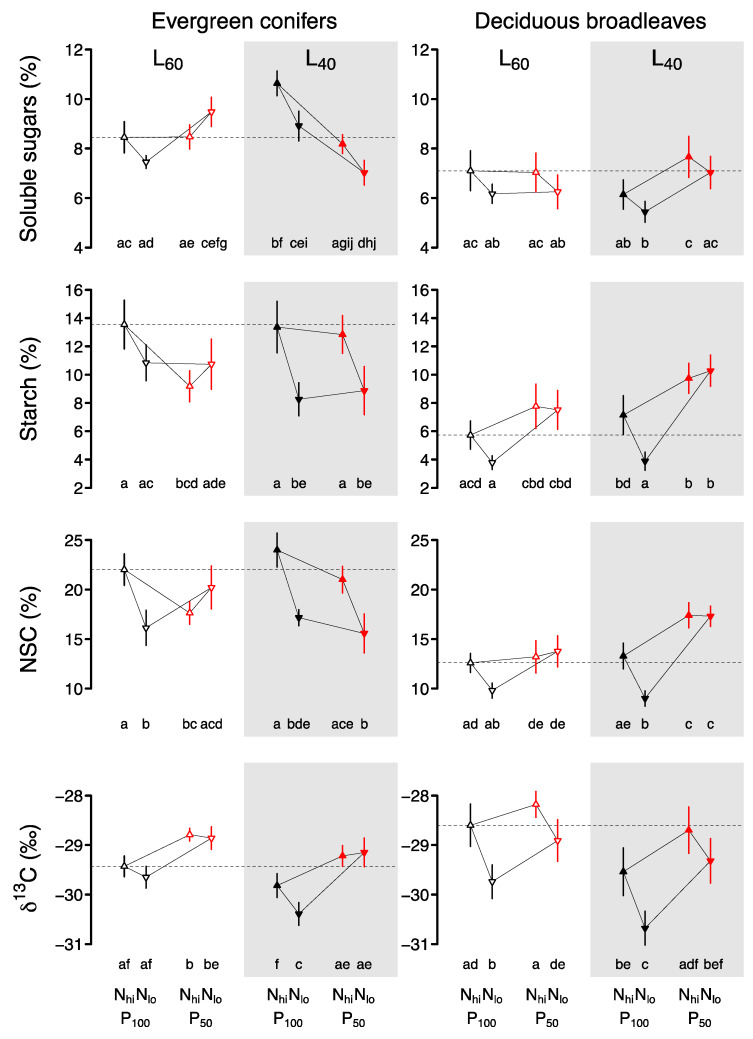
Foliar soluble sugar, starch, total non-structural carbon (NSC) concentration, and carbon isotope fractionation (δ^13^C; means ± SE) of evergreen conifer and deciduous broadleaved seedlings grown for three years under fully crossed combinations of ambient (P_100_, black symbols) or reduced precipitation (P_50_, red symbols), high (L_60_, open symbols) or low PAR (L_40_, filled symbols), and high (N_hi_, upwards triangle) or low (N_lo_, downwards triangle) soil nutrient availability. The dashed line indicates foliar traits under optimal resource availability, i.e., L_60_ × P_100_ × N_hi_. Different letters below the means indicate significant differences between treatment combinations within species group (Tukey adjusted least square means).

Foliar soluble sugars (SS), starch (St), and non-structural carbohydrate concentrations (NSC) of EC showed complex responses to the interactive effects of precipitation, shading, and nutrients (Table 1, Figure 2). Under high PAR (L_60_), the amount of precipitation and nutrients did not affect SS, but low PAR (L_40_) combined with low precipitation (P_50_) and low nutrients (N_lo_) led to lower concentrations of SS. Similarly, under high PAR (L_60_), nutrients had no effect on St, but under low PAR, low nutrients (L_40_ × N_lo_) reduced St. Interestingly, under high PAR and high nutrient availability, low precipitation significantly decreased St (L_60_ × P_50_ × N_hi_) compared to ambient precipitation (L_60_ × P_100_ × N_hi_), but no such effect was observed for seedlings grown under low PAR or low nutrient availability. As St was much more abundant than SS, the NSC responses to precipitation, shading, and nutrients were similar to those of St.

Foliar carbon fractionation (δ^13^C) of EC responded very strongly to precipitation, and to a lesser degree to shading (Table 1). δ^13^C was higher in needles grown under low precipitation (P_50_) compared to ambient precipitation (P_100_) in all shading and nutrient treatments, and foliar δ^13^C was generally smaller in low light (L_40_) than high light (L_60_) environments (Figure 2).

### 2.2. Deciduous Broadleaves

SLA of deciduous broadleaf (DB) seedlings was affected by shading and nutrients, but not by precipitation (Table 2). Foliage grown under either low nutrient (N_lo_) or low PAR (L_40_) had both higher SLA compared to high nutrient (N_hi_) or high PAR (L_60_), respectively (Figure 1). But, the cumulative effect of low nutrient and low PAR was weak so that foliage grown under low PAR and low nutrient (L_40_ × N_lo_) was only slightly higher than foliage grown under low light and high nutrient (L_40_ × N_hi_) and not different from foliage grown under high PAR and low nutrient (L_60_ × N_lo_). The C/N ratio of DB was affected by both precipitation and nutrients (Table 2). It was higher in foliage from low nutrients (N_lo_) in all shading and precipitation treatments (Figure 1). Low precipitation, however, reduced the C/N ratio, but only in foliage grown under high nutrients (P_50_ x N_hi_). 

SS, St, and NSC of DB responded to all three treatments (Table 2). Overall, SS was lower in foliage grown under low nutrients (N_lo_), compared to high nutrients (N_hi_), but post-hoc tests showed no significant differences between groups (Figure 2). In seedlings grown under low PAR, the low precipitation (L_40_ × P_50_) led to higher SS compared to low PAR and high precipitation (L_40_ × P_100_). Foliar St showed a strong response to precipitation, and was significantly higher in foliage under combined low precipitation and low nutrients (P_50_ × N_lo_) than high precipitation and low nutrients (P_100_ × N_lo_) in both shading treatments. A similar tendency seems to exist in seedlings grown under high nutrient availability, but group comparisons were not significant. NSC responded to the treatments similarly as St, except that in foliage from low precipitation, the low PAR increased NSC (P_50_ × L_40_) compared to foliage from high PAR (P_50_ × L_60_).

δ^13^C was severely affected by all three treatments (Table 2). Low precipitation generally led to higher δ^13^C, whereas low PAR decreased it, but differences were strongest in seedlings from low nutrient treatments. Foliage grown at low nutrient concentration had a consistently lower δ^13^C compared to high nutrients, except in seedlings grown at low PAR and low precipitation (L_40_ × P_50_). Overall, foliage from low PAR, low nutrient, and high precipitation had the lowest δ^13^C, whereas foliage from high PAR, high nutrient, and low precipitation had the highest δ^13^C (Figure 2).

## 3. Discussion

### 3.1. Foliar Responses of Evergreen Conifers and Deciduous Broadleaves

In both species groups, the foliar traits SLA and C/N ratio reacted primarily to altered nutrient availability and precipitation, and less to light conditions. According to their contrasting life-history strategy, the two species groups showed opposite reactions of SLA to nutrient deficiency: low N/P/K limited the foliar growth of DB both in terms of leaf mass and leaf area, i.e., the seedlings produced smaller and thinner leaves (Figure S1). This means that the whole plant has to maintain a smaller canopy in the advent of drought and may thus better resist drought [11]. In contrast, under nutrient shortage, EC slightly increased the foliar area while needle dry weight remained constant (Figure S1), which is in accordance with the higher tolerance to low resource availability of a “slow” life-history strategy [34]. Another marked difference between the two species group is that SLA of EC was independent of light availability, whereas SLA of DB increased under low PAR combined with high nutrient and water availability. As long as neither nutrients nor water were limiting (P_100_ × N_hi_), DB thus responded to low PAR according to “optimal partitioning”, i.e., with higher investments in the organs capturing the most limiting resource, light [18,19,48]. Interestingly, while plants exposed to water stress have been shown to reduce SLA [45], the precipitation treatment did not affect DB but increased SLA of EC under high light availability due to a reduction in foliar dry weight (Figure S1). Responses of foliar C/N ratio also differed between the two species groups (Figure 1, Table 1; Table 2). In particular, DB showed a very strong response of C/N to nutrient availability (Figure 1) with a much lower C/N under high nutrients compared to low nutrients. The foliar N content is related to photosynthetic efficiency [15,49]. Thus, a low C/N ratio indicates a higher investment in proteins of the photosynthetic machinery. The strong response of DB thus shows a high potential of this species group to efficiently use available soil nutrients to increase foliar N, potentially enhancing photosynthetic efficiency. In contrast, EC retains a relatively constant but high C/N ratio, which suggests large carbon investments in cell walls, cuticula, and other structures related to the mitigation of evaporation, protection against herbivory [50,51], and long leaf life span [29]. Such characteristics enable trees to grow opportunistically as suggested by a “slow” life-history strategy, but limit their potential to enhance photosynthetic efficiency under high nutrient conditions. 

The foliar carbon isotope fractionation (δ^13^C) responded strongly to precipitation, shading, and nutrients in both species groups (Table 1 and Table 2). Most notably, δ^13^C was higher in seedlings exposed to low precipitation and was lower in seedlings from mesocosms with both low PAR or low nutrient availability (Figure 1). The effects differ in strength between the two species groups, but they show the same general trend. The δ^13^C of the seedlings can be used as an integrative measurement of long-term foliar CO_2_ exchange and indicate stomatal limitation of photosynthesis [52]. A higher, i.e., less negative, δ^13^C translates to a higher assimilation rate in relation to leaf conductance (A/*g*_S_) and to a higher water-use efficiency (WUE; [53]). Foliar δ^13^C is driven by the draw-down of intercellular CO_2_ (c_i_) via assimilation and the supply of atmospheric CO_2_ (c_a_) through the stomata. Thus, a high δ^13^C in seedlings grown under dry conditions shows that water stress induced low g_S_, restricting assimilation of seedlings in both species groups (Figure 2). Conversely, the small δ^13^C of seedlings grown under low irradiance indicates a smaller draw-down of c_i_ as assimilation was limited under shady conditions. Similarly, the low δ^13^C of seedlings grown under nutrient shortage indicates that low soil nutrient availability reduced assimilation. The photosynthesis machinery consists of proteins that are nitrogen-consuming [54,55]. Consequently, nutrient shortage might result in a deficiency of these proteins and therefore constrict photosynthesis. Interestingly, nutrient availability had only minor effects on δ^13^C in evergreen conifers, except under conditions of high precipitation and low PAR, when low nutrients reduced δ^13^C (Figure 2). This indicates that photosynthesis of EC responded less to changes in soil nutrient availability, again concurring with a “slow” resource strategy. 

The stomatal and biochemical limitation of photosynthesis affected soluble sugars (SS), starch (St), and total non-structural carbohydrate (NSC) concentrations in the foliage of both EC and DB. We observed slightly reduced SS in the foliage of DB seedlings grown in low nutrient soils, while low nutrients only affected SS of EC needles in combination with low light conditions. This is in accordance with Schönbeck et al. [13] who found no reaction of nutrient availability on foliar gas exchange and NSC concentrations of *P. sylvestris* in a high light environment. As foliar δ^13^C indicated a limited photosynthetic capacity of seedlings grown under low nutrient availability, it is possible that low SS resulted from carbon source limitation. On the other hand, SS are preferentially allocated to roots in drought stressed seedlings in order to maintain water transport [56,57]. Similarly, SS may have been preferentially allocated to roots in order to capture the limiting soil nutrients. Low light availability further reduced foliar δ^13^C in both species groups, indicating that low light availability restricted the draw-down of c_i_, thereby limiting carbon uptake [58]. But in contrast to seedlings from low nutrient treatments, low light availability did not reduce SS, St, or NSC concentrations (Figure 2). A high δ^13^C can therefore not be directly translated to reduced carbon pools in the foliage of tree seedlings. Precipitation had contrasting effects on foliar SS, St, and NSC, depending on the species group: while water stress resulted in high SS, St, and NSC in DB, the response of EC depended on light conditions (Figure 1). In order to draw conclusions regarding implications of water stress for the plant carbon status of the two species groups, additional information on NSC in other plant compartments such as stems and root wood is needed. The similar δ^13^C and contrasting NSC responses of BD and EC to precipitation strongly indicate that the two species groups differ in the way they process and allocate carbon under drought. While low foliar NSC of EC under drought might indicate carbon source limitation [59], high foliar NSC of DB might result from hampered transport to sink organs [56,60,61,62,63]. 

### 3.2. Life-History Strategies as the Key to Foliar Responses to Environmental Changes

We found that EC and DB respond differently to precipitation, nutrients, and light availability in several foliar traits, which can be explained by contrasting life-history strategies of the two species groups. DB species showed “fast” foliar traits with a relatively high SLA and low C/N ratio, whereas EC species’ traits followed a typical “slow” functionality with a low SLA and high C/N ratio [22,29,44]. In general, DB leaf traits allow seedlings to achieve higher relative growth rates than EC species [63,64]. As hypothesised, DB responded stronger to altered environmental conditions, particularly to excess nutrient supply (Figure 1). High investments of DB in foliar N (low C/N ratio) and leaf area under high soil nutrient and light availability account for high gas exchange rates (less negative δ^13^C), resulting in higher growth rates. On the other hand, low nutrient and light availability led to a higher foliar C/N ratio and lower SLA, which ultimately reduces productivity. 

“Slow” EC are less plastic in their foliar traits, in particular with respect to soil nutrient availability (Figure 1 and Figure 2). Only the combination of low light and low nutrient availability resulted in the lower δ^13^C that indicates limited assimilation. This shows that EC have a wide stress tolerance to low light and low nutrient status, as hypothesized. Evergreen species sustain their leaves across the winter or dry season, which necessitates a lower activity in the cold or dry period to resist and tolerate low temperature or drought [65]. Therefore, they need to be able to assimilate at low water availability by, e.g., small leaves, low SLA [66,67,68]. Foliar habit is therefore an adaptive strategy of plants to tolerate, resist, and survive under harsh environmental conditions [46,69,70]. While the “slow” strategy enhances survival under low resource availability, the low growth rates associated with this strategy compromise a species’ competitive ability in high resource environments [22]. We are aware that competition might have interacted with effects of water, light, and nutrient availability in our experiment, especially in EC and under high resource conditions. However, except in commercial plantations, seedling establishment in Central European lowland forests occurs in the presence of other species. Consequently, our experimental set-up mirrors natural conditions more closely than pot experiments, for instance. Experiments under near natural conditions are important for predictions of potential shifts in composition and structure of forest communities under future climatic conditions. 

## 4. Materials and Methods 

### 4.1. Plant Material and Experimental Design

We measured foliar traits on 3-year-old plants of broadleaved *Acer pseudoplatanus* L., *Fagus sylvatica* L., *Quercus robur* L. and *Quercus petraea* Liebl., and coniferous *Abies alba* Mill., *Picea abies* (L.) H. Karst., *Pinus sylvestris* L. and *Pseudotsuga menziesii* (Mirb.) Franco. These species are currently the main constituents of temperate Central European lowland forests. While *P. menziesii* was introduced from western North America to Europe in the 19th century and is now extensively planted across Central and Western Europe, all other species are natives. The eight species were grown from seeds (Table S1) in mesocosms located in a common garden at WSL, Switzerland (47°21′38.3″ N, 8°27′16.6″ E; 550 m a.s.l.; mean annual temperature 9.3 °C, mean annual precipitation 1134 mm, MeteoSwiss station Fluntern, 1981–2010). The mesocosms (1 m × 1 m surface, 0.5 m deep) were filled with a mixture of quartz sand, fibric peat, expanded schist, and pumice. They were arranged in a split-split-plot design with *precipitation* x *light* as whole-plot factors, *nutrients* as the split-plot factor, and *species* as the split-split-plot factor. Precipitation was manipulated from May–October with throughfall reduction shelters that allowed for 100% or 50% of ambient rainfall (P_100_, P_50_; Figure S2). To ensure constant germination and seedling establishment across treatments, all mesocosms were regularly irrigated from April (seed sowing) to mid-July 2016. The total precipitation sums from May–October amounted to 729 mm (P_100_) and 607 mm (P_50_) in 2016, 619 mm and 294 mm in 2017, and 672 mm and 340 mm in 2018, respectively. During periods of extreme drought, mesocosms were watered manually so that the plants received the long-term average precipitation of the region in the P_100_ treatment (50% in the P_50_ treatment). All mesocosms were shaded from May–October and received either 38.8 ± 0.021% (mean ± SE; L_40_; Figure S2a,b) or 58.0 ± 0.022% (L_60_; Figure S2c,d) of the photosynthetically active radiation (PAR_mesocosm_/PAR_ambient_*100; measured on three days in August and September 2018). The soil substrate was designed to contain a low amount of *nutrients* (0.0014/<0.001/0.018 g N/P/K m^−2^) so that we were able to manipulate soil nutrients by adding Gesal Floranid slow release lawn fertilizer twice a year in April and August. Total nutrient additions amounted to 4.1/1.3/3.7 and 17.3/5.7/17.1 g N/P/K m^−2^ in the low (N_lo_) and high (N_hi_) nutrient treatment in 2016, and 6/1.5/2.4 and 24.0/6.0/9.6 g N/P/K m^−2^ in 2017 and 2018. We increased nutrient additions in the second and third growing season to account for higher plant biomass.

Each mesocosm was divided into 48 sowing quadrats of 10 cm × 10 cm in which 3–10 seeds were sown in April 2016 (six sowing quadrats per species; seed origin s. supplementary Table S1). In October 2016, 1–2 seedlings per sowing quadrat were harvested, and the number of seedlings was reduced to equal numbers (3 for EC, 2 for DB) after leaf out in spring 2017. After the second harvest in October 2017, one seedling per sowing quadrat remained for the third growing season in 2018. Seedling emergence was extremely low in *F. sylvatica* so that in September 2016, seedlings from the same seed lot grown as a back-up in the nursery were planted in the empty quadrats.

### 4.2. Foliar Traits

We measured foliar traits on a random subset of two individuals per species and mesocosm, i.e., a total of six individuals per treatment combination (6 individuals × 8 species × 2 levels of precipitation × 2 levels of light × 2 levels of soil nutrients = 384 individual seedlings). Ten healthy, fully expanded fresh leaves per individual were collected at random, immediately stored in paper bags in a cool box, and kept in the fridge at −20 °C until analysis. All leaf traits were measured following standardized protocols [71]. To calculate foliar area (cm^2^), a random subset of (i) 3–4 leaves per DB individual and (ii) 8 needles per EC individual was scanned at 600 dpi using the SliverFast 8 software. The image processing software Pixstat v.1.2.0.0 was then used to calculate foliar area of DB, while needle width and needle length were calculated for EC. Foliar area of EC was calculated as needle width × needle length [72]. Specific leaf area (SLA, cm^2^ g^−1^) was calculated as the leaf area per unit dry weight. After scanning, all leaves of the same species within a mesocosm were pooled, oven-dried (65 °C or 72 h) and ground with a Retsch MM400 ball mill using steel containers. We then measured leaf non-structural carbohydrates (NSC), i.e., soluble sugars (SS) and starch (St), of the pooled sample following the protocol described in Schönbeck et al. [13]. Total leaf C and N content and δ^13^C were also analyzed according to Schönbeck et al. [13]. The isotopic ratios in all samples were expressed in δ notation (‰) relative to the international standard Vienna Pee Dee Belemnite (VPDB). C and N contents were assessed as a percentage of dry weight.

### 4.3. Data Analyses

Effects of species, along with water, light, and nutrient availability, on foliar morphological (SLA, C/N ratio, foliar area and weight) and foliar biochemical traits (soluble sugars, starch, non-structural carbohydrates, δ^13^C) were analyzed separately for EC and DB. Linear mixed effects models were fitted using R 3.4.0 and the lmer function of the lme4 package with blocks as random effect, and precipitation, light, nutrients, and species, respectively, as fixed effects. Species were considered as nested within blocks. As the lmer function does not provide *P* values for the fixed effects, *P* values were calculated using the lmerTest package.

Data available from the Dryad Digital Repository https://doi.org/10.5061/dryad.zkh189376. 

## 5. Conclusions

Seedlings of DB and EC showed contrasting responses of their foliar traits to resource availability, indicating fundamental differences in the way the two species groups regulate their uptake, metabolism, and allocation of the available water, soil nutrients, and carbon. Our results support the notion that species with “fast” functional traits, such as DB, show higher trait plasticity than “slow” species [73]. High trait plasticity may confer tolerance to resource fluctuation by enhancing resource-capture efficiency [74]. Although at short timescales, EC are able to adjust their foliar physiology to drought [59], a slow response to long-term changes in resource availability makes evergreen species vulnerable to environmental change [75]. To cope with environmental change, EC species might therefore migrate rather than acclimate in a changing world [76].

## Figures and Tables

**Table 1:** Linear mixed model results of the effects of species (S), precipitation (P), shading (L), and nutrients (N) on the specific leaf area (SLA), foliar C/N ratio, foliar soluble sugars concentration, foliar starch concentration, foliar total non-structural carbohydrate concentration (NSC), and foliar carbon isotope fractionation (δ13C) of evergreen conifers.

Conifers	SLA		C/N		Sugars		Starch		NSC		δ13C	
	*F*	*P*	*F*	*P*	*F*	*P*	*F*	*P*	*F*	*P*	*F*	*P*
Species (S)	54.20	<0.001	14.59	<0.001	5.57	0.0018	23.98	0.0010	10.59	<0.001	13.86	0.0041
Nutrients (N)	7.85	0.0057	3.80	0.0559	4.57	0.0363	13.70	<0.001	16.29	<0.001	2.29	0.1362
Precipitation (P)	6.74	0.0103	0.88	0.3525	2.87	0.0952	2.95	0.0917	1.58	0.2130	40.32	<0.001
Light (L)	0.72	0.3972	1.79	0.1861	0.43	0.5147	0.43	0.5143	0.20	0.6538	12.80	<0.001
S × N	0.13	0.9391	0.27	0.8475	0.39	0.7635	3.31	0.0266	1.06	0.3735	2.24	0.0932
S × P	0.24	0.8689	0.98	0.4096	1.35	0.2658	1.26	0.2960	0.97	0.4132	1.31	0.2808
N × P	0.45	0.5044	8.39	0.0052	3.62	0.0615	0.69	0.4090	6.50	0.0132	2.33	0.1329
S × L	0.46	0.7110	0.75	0.5287	1.85	0.1467	0.81	0.4923	0.66	0.5798	1.27	0.2942
N × L	2.51	0.1149	0.09	0.7666	4.63	0.0353	8.97	0.0041	5.38	0.0237	0.19	0.6606
P × L	4.45	0.0366	1.76	0.1901	22.53	<0.001	2.25	0.1392	1.22	0.2743	0.62	0.4362
S × N × P	1.70	0.1700	0.86	0.4650	0.87	0.4610	0.55	0.6502	1.60	0.1979	0.29	0.8347
S × N × L	2.10	0.1025	1.25	0.3001	0.96	0.4173	0.59	0.6247	0.82	0.4874	0.34	0.7994
S × P × L	0.04	0.9898	0.13	0.9414	0.51	0.6769	0.29	0.8292	0.95	0.4241	1.44	0.2407
N × P × L	0.34	0.5627	0.26	0.6113	1.16	0.2862	1.03	0.3147	3.34	0.0723	0.86	0.3581
S × N × P × L	1.42	0.2401	2.65	0.0568	0.33	0.8057	0.02	0.9948	0.33	0.8054	0.60	0.6173

**Table 2:** Linear mixed model results of the effects of species (S), precipitation (P), shading (L), and nutrients (N) on the specific leaf area (SLA), foliar C/N ratio, foliar soluble sugars concentration foliar starch concentration, foliar total non-structural carbohydrate concentration (NSC) and foliar carbon isotope fractionation (δ13C) of deciduous broadleaves.

Broadleaves	SLA		C/N		Sugars		Starch		NSC		δ^13^C	
	*F*	*P*	*F*	*P*	*F*	*P*	*F*	*P*	*F*	*P*	*F*	*P*
Species (S)	7.81	0.0167	19.47	0.0018	15.74	0.0011	2.20	0.1927	0.70	0.5573	59.43	<0.001
Nutrients (N)	20.50	<0.001	140.01	<0.001	5.41	0.0236	2.83	0.0979	3.65	0.0608	30.49	<0.001
Precipitation (P)	1.81	0.1805	13.23	<0.001	6.30	0.0150	27.53	<0.001	27.90	<0.001	27.73	<0.001
Light (L)	8.41	0.0043	3.44	0.0689	0.06	0.8130	4.74	0.0337	5.42	0.0232	18.33	<0.001
S × N	2.56	0.0569	14.64	<0.001	5.33	0.0026	0.71	0.5472	0.40	0.7565	1.29	0.2872
S × P	0.02	0.9968	5.63	0.0019	5.06	0.0036	5.05	0.0036	3.45	0.0219	1.70	0.1755
N × P	0.43	0.5122	3.59	0.0632	0.03	0.8727	3.66	0.0610	5.23	0.0256	1.95	0.1680
S × L	1.25	0.2952	1.63	0.1926	0.64	0.5947	0.93	0.4317	0.58	0.6313	1.39	0.2548
N × L	1.96	0.1636	0.69	0.4107	0.11	0.7421	0.02	0.8763	0.39	0.5367	0.03	0.8674
P × L	0.33	0.5674	0.09	0.7592	6.55	0.0132	1.46	0.2323	6.54	0.0130	2.07	0.1552
S × N × P	0.61	0.6107	2.19	0.0989	1.37	0.2617	1.60	0.2006	0.13	0.9450	1.59	0.2015
S × N × L	1.67	0.1765	1.24	0.3029	1.94	0.1331	0.71	0.5496	0.66	0.5791	1.21	0.3148
S × P × L	1.03	0.3825	0.41	0.7436	0.19	0.9055	0.49	0.6905	0.50	0.6808	1.19	0.3215
N × P × L	0.03	0.8570	0.00	0.9889	0.02	0.8761	0.47	0.4978	0.02	0.8992	0.02	0.8763
S × N × P × L	3.00	0.0327	1.21	0.3150	1.89	0.1416	1.46	0.2342	0.06	0.9794	0.12	0.9502

## Supplementary Materials

The following are available online at https://www.mdpi.com/2223-7747/9/5/621/s1, Figure S1: Leaf area and dry weight, foliar C and N content, Figure S2: The experimental set-up, Table S1: Seed material used in the common garden experiment.
