# Supplementary material for: Contrasting Leaf Trait Responses of Conifer and Broadleaved Seedlings to Altered Resource Availability Are Linked to Resource Strategies
Source: Plants (Basel). 2020 May 13;9(5):621. doi: 10.3390/plants9050621 (PMC7285355; doi:10.3390/plants9050621)
Supplement: Supplementary file 1 [file plants-09-00621-s001.pdf]

Supplementary Material

Table S1. Seed material used in the common garden experiment.

**Species**	**Provenance**	**Latitude (°N)**	**Longitude (°E)**	**Elevation (m a.s.l.)**	**Year of seed collection**
**Evergreen conifers**					
Abies alba	Leuk, Switzerland	46.33	7.64	1250	2015
Picea abies	Bremgarten, Switzerland	47.36	8.31	425	2015
Pinus sylvestris	Leuk, Switzerland	46.31	7.61	630	2016
Pseudotsuga menziesii var. menziesii	Washington, USA	46.76	-122.06	435	2013
**Deciduous broadleaves**					
Acer pseudoplatanus	Arni, Switzerland	47.31	8.43	575	2014
Fagus sylvatica	Hausen, Switzerland	47.23	8.57	705	2014
Quercus petraea	Erlach, Switzerland	47.03	7.09	380	2015
Quercus robur	Aristau, Switzerland	47.30	8.38	453	2015

Zhang et al. Contrasting leaf trait responses of conifer and broadleaved seedlings to altered resource availability are linked to resource strategies

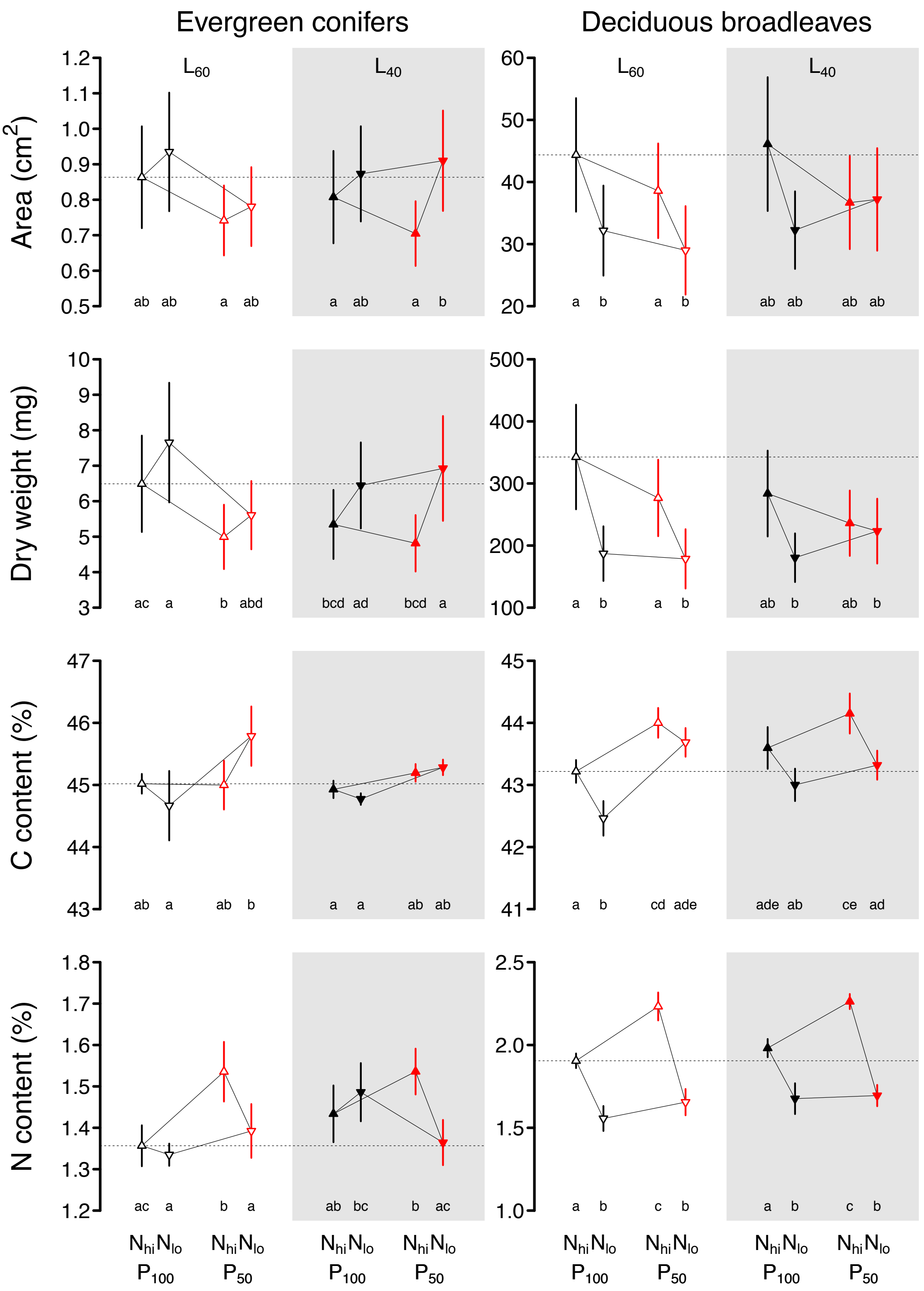

Figure S1. Area, dry weight, C and N content (means ± SE) of the foliage of evergreen conifer and deciduous broadleaved seedlings grown for three years under fully crossed combinations of ambient (P_{100}, black symbols) or reduced (P_{50}, red symbols) precipitation, high (L_{60}, open symbols) or low (L_{40}, filled symbols) PAR, and high (N_{hi}, upwards triangle) or low (N_{lo}, downwards triangle) soil nutrient availability. The dashed line indicates foliar traits under optimal resource availability, i.e. L60 x P100 x Nhi. Different letters below the means indicate significant differences between treatment combinations within species group (Tukey adjusted least square means).

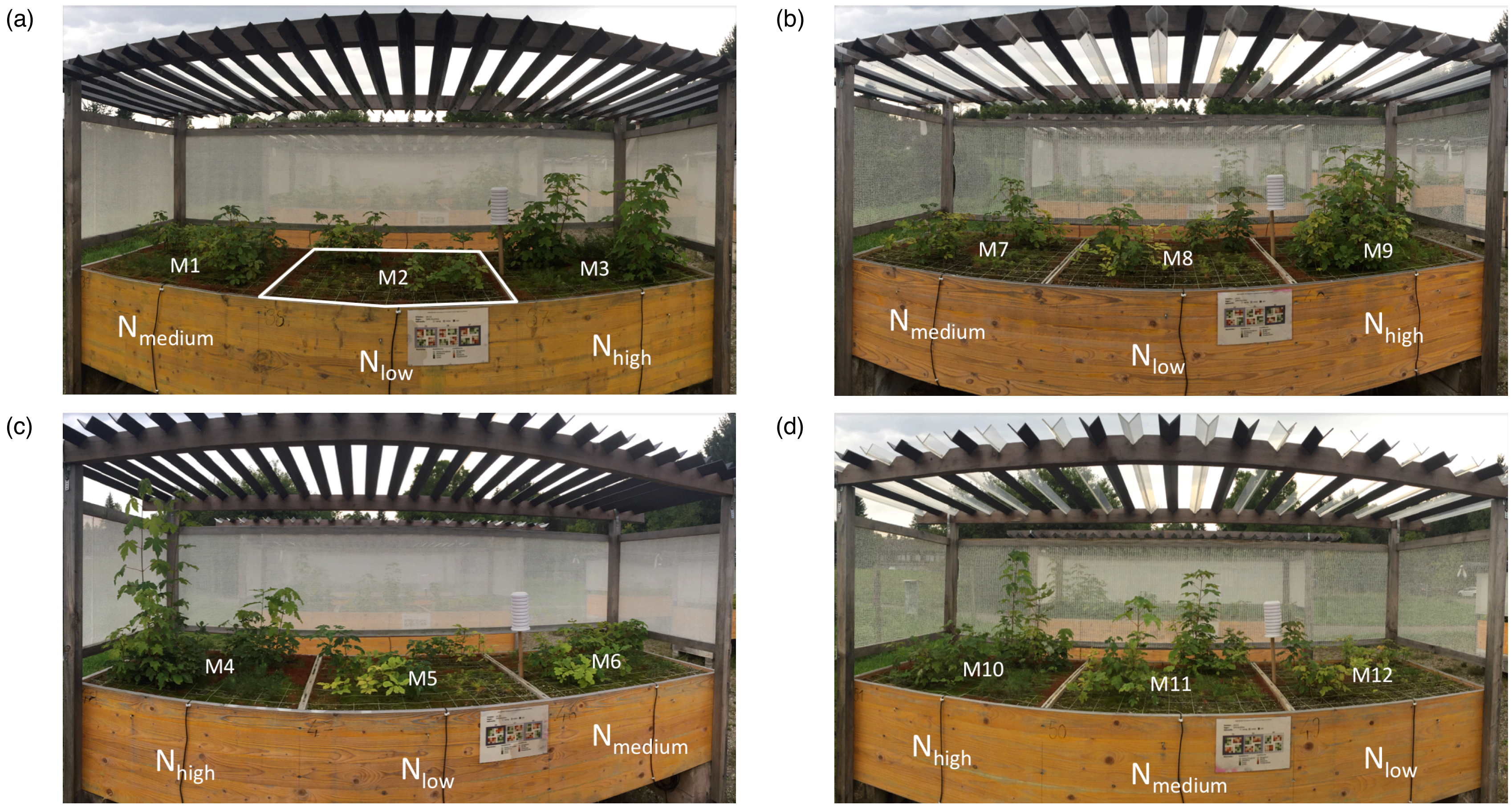

Figure S2. The experimental set-up consisted of 36 mesocosms (M1–M12) of 1 m x 1 m x 0.5 m arranged in sets of three. In order to manipulate water availability, each triplet was covered either with (a,b) a roof that allowed 100% precipitation throughfall (P_{100}: V-shaped plastic channels mounted downwards; M1–M3, M7–M8), or (c,d) a roof that removed 50% of the ambient precipitation (P_{50}: V-shaped channels that cover 50% of the mesocosm surface; M4–M6,M10–12). Light conditions were manipulated with the colouring of the channels (either all or 50% of the channels covered with black plastic foil impenetrable to light) combined with shade cloth of varying mesh size attached to the east, south and west of each mesocosm-triplet: (a,c) medium shade corresponding to 38.8±0.021 % (mean ± SE) of photosynthetically active ration (PAR; L_{40}); (b,d) light shade with 58.0 ± 0.022 % PAR. Different amounts of nutrients were added twice a year to the three mesocosms in a triplet (N_{low}, N_{medium} N_{high}). Leaf traits were only studied in the N_{low} and N_{high} mesocosms.
